# Integrating assisted tele-psychiatry into primary healthcare in Goa, India: a feasibility study

**DOI:** 10.1017/gmh.2021.47

**Published:** 2022-02-03

**Authors:** Ankur Garg, Ravindra Agrawal, Richard Velleman, Anil Rane, Sheina Costa, Devika Gupta, Ethel Dsouza, Abhijeet Jambhale, Akshada Sabnis, Godwin Fernandes, Urvita Bhatia, Abhijit Nadkarni

**Affiliations:** 1 Sangath, Porvorim, Goa, India; 2 Antarman Centre for Psychosocial Wellbeing, Panjim, Goa, India; 3 Manipal Hospital, Panaji, Goa, India; 4 University of Bath, Bath, UK; 5 Institute of Psychiatry & Human Behaviour, Bambolim, Goa, India; 6 Manovikas English Medium School, Margao, Goa, India; 7 Department of Population Health, London School of Hygiene & Tropical Medicine, Centre for Global Mental Health, London, UK; 8 Department of Psychology, Health and Professional Development, Oxford Brookes University, Headington Rd, Headington, Oxford OX3 0BP, UK

**Keywords:** Tele-psychiatry, common mental disorders, primary care, India

## Abstract

**Background:**

Tele-psychiatry is an increasingly acceptable and feasible platform to deliver mental health care with the potential to increase access to care in low-resource settings. We aim to examine the acceptability and preliminary impact of the delivery of assisted tele-psychiatry services in primary healthcare settings in Goa, India.

**Methods:**

Before-after uncontrolled treatment cohort study. In total, 161 adults with either a mental or alcohol use disorder were provided tele-consultation by psychiatrists through a customised video conferencing platform, along with medication or counselling (via trained lay counsellors) or both as needed. Data on socio-demographics, clinical outcomes and process indicators were collected at baseline and 3 months post-baseline. Paired *t* tests were used to assess clinical outcomes pre- and post-treatment using the General Health Questionnaire-12 (GHQ-12) and World Health Organisation Disability Adjustment Schedule (WHODAS) 2.0, and logistic regression was used to find associations between changes in these scores and various factors.

**Results:**

The most common diagnosis was depression (35%). Post-treatment, there was a significant reduction in both GHQ-12 and WHODAS 2.0 scores. Participants showed high satisfaction with the tele-psychiatry services and technology platform. Improvement in GHQ-12 score was associated with being employed [OR 8.74 (1.92–39.75, *p* = 0.005)] and being a homemaker [OR 6.42 (CI 1.61–25.57, *p* = 0.008)].

**Conclusion:**

Treatment of mental disorders through a tele-psychiatry platform appears to be highly acceptable and is associated with improved clinical outcomes. Considering its potential for scalability, a model of assisted tele-psychiatry integrated into primary care can be an important strategy to increase access to mental healthcare in low-resource settings.

## Background

Mental and substance use disorders affect 16% of the global population (Rehm and Shield, 2019). Globally, mental disorders are the second leading contributors to the years lived with disability and the sixth largest contributor to disability-adjusted life years (Sagar *et al*., 2020). Mental disorders substantially reduce the quality of life, productivity, social functioning and workforce participation among patients, their family members and society; with a projected global economic loss of $16 trillion by 2030 (Patel *et al*., 2018; United for Sight, 2020). In India, 14% (~197 million) of the population experiences a mental disorder of some severity (Sagar *et al*., 2020).

India's spend on mental health is only 2% of the total health budget, which in turn is only 5% of its GDP (Jacob *et al*., 2007). Furthermore, India's mental health workforce (per 100 000 population) is much smaller compared to high-resource countries such as the United States (US), e.g. psychiatrists (0.3 in India *v.* 10.54 in the US), nurses (0.8 *v.* 4.28), psychologists (0.07 *v.* 29.86) and social workers (0.06 *v.* 60.34) (World Health Organization, 2016). Additionally, a majority of mental health services are disproportionately situated in urban areas (World Health Organization, 2005) and most patients are forced to travel long distances to access mental health care (Lahariya *et al*., 2010).

Such shortage of trained human resources and their geographical inaccessibility are key barriers resulting in an overall treatment gap of 83% for any mental disorder in India (Murthy, 2017). Furthermore, the implementation of the Government of India's flagship District Mental Health Programme (DMHP) (van Ginneken *et al*., 2014; NHP Admin, 2019) has been suboptimal and more than 20 years after its launch, its reach is limited to only 189 of the 626 intended districts across the country (Murthy and Sciences, 2018).

India's National Mental Health Policy (NMHP) 2014 emphasised decentralised delivery of mental health services at the primary care level as the most practical and feasible solution to address the treatment gap (Ministry of Health & Family Welfare, 2014; van Ginneken *et al*., 2014). Despite the inherent advantages of such an approach including reduction in travelling distance and better follow-up opportunities (Srinivasa Murthy, 2011), its implementation has failed due to various factors including, most importantly, the shortage of skilled mental health professionals (Goel, 2011; van Ginneken *et al*., 2014).

While expanding the traditional service delivery infrastructure is limited by implementation challenges and has a long gestation period (Malhotra *et al*., 2013), technological innovations provide several opportunities to overcome the barriers faced by conventional healthcare delivery systems. Tele-psychiatry (i.e. psychiatric consultation through videoconferencing) is a promising and scalable long-term option in areas with limited resources (Simpson *et al*., 2001), especially to deliver care to underserved populations (Hilty *et al*., 1999) and to those living in remote areas (De Las Cuevas *et al*., 2006).

In low-resource settings such as India, which has a poor mental health infrastructure with a near absence of access to mental health care in rural settings, there is a need to examine the use of tele-psychiatry as a viable and feasible real-world option. Although tele-psychiatry has been used sporadically in India, its formal integration into primary care has not yet been systematically attempted in the country. Additionally, the use of an ‘assisted tele-psychiatry’ model (where patients are assisted by lay counsellors to connect to the psychiatrist, and to undertake other tasks such as scheduling of sessions, data collection, session facilitation, home visits, delivering counselling sessions, etc.) (Atta-ur-Rahman and Basheer Ahmed, 2019) has particular relevance to India, given the low levels of digital literacy rates, especially in rural areas (Team SPRF, 2020).

In the formative phase of the IMPACT (IMproving ACcess through Tele-psychiatry) project, we examined the existing global evidence on tele-psychiatry services and conducted in-depth interviews with a range of stakeholders (including psychiatrists, patients and caregivers) to understand the process, synergies and potential limitations of providing tele-psychiatry in the community. This led to the development of a contextually appropriate ‘assisted tele-psychiatry’ package for use in primary care. This paper describes the implementation of that package to test its integration in the primary care setting, its acceptability to patients and its preliminary impact.

## Methodology

### Study design

Before-after uncontrolled treatment cohort.

### Setting

Goa, on the west coast of India, with a population of approximately 1.4 million (Census, 2011). Mental healthcare in Goa is provided by both public (government) and private healthcare providers. Public mental healthcare is provided mainly through a large tertiary care teaching hospital in the state's capital and two district hospitals. Although the state does have rural coverage of healthcare services in the form of primary/community health centres (PHCs/CHCs), there is limited and infrequent availability of mental health services through the DMHP. Furthermore, although in many high-income countries, primary care doctors and other staff provide at least some mental health care (e.g. prescription of anxiolytics and antidepressants), that is not expected as part of the PHC doctor's role in Goa. Private mental health care is largely restricted to urban areas and generally affordable only to the affluent. Mental health services in the community are inadequate, with the majority of the population having poor access and those living in remote areas being especially underserved.

We recruited participants from the outpatient department of one community health centre (expected to cater to a population of 120 000) and two primary health centres (each expected to cater to a population of 30 000) in North Goa. In South Goa, we recruited from one public trust hospital (i.e. a general hospital) primarily serving the staff and family members of the Port Trust and one rural community (catchment population of 14 795). The sites were selected based on practical considerations such as local government permissions.

### Sample

#### Inclusion criteria

Adults (⩾18 years) with one or more of the following: (a) probable common mental disorder defined as a score of 4 and above on the Global Health Questionnaire (GHQ-12) (Goldberg and Blackwell, 1970), (b) alcohol use disorder defined as a score of >15 on the Alcohol Use Disorders Identification Test (AUDIT) (Babor *et al*., 1992), and (c) any other mental disorder as identified by the lay counsellor or psychiatrist (based on their clinical judgement).

#### Exclusion criteria

Patients: (a) requiring immediate medical attention, (b) not planning to reside in the study catchment area for the next 6 months, or (c) unable to speak in either English or one of the three vernacular languages – Hindi, Konkani or Marathi.

### Delivery agents and training

The tele-consultation sessions were provided by one of two psychiatrists with medical degrees, specialist psychiatric training and more than 5 years of experience in clinical practice. They were assisted by a group of four lay counsellors: non-specialist workers who were educated to at least senior secondary level and had subsequently been trained to deliver basic counselling and evidence-based psychological treatment packages for depression [Healthy Activity Program (HAP)] (Patel *et al*., 2017) and alcohol use disorders [Counselling for Alcohol Problems (CAP)] (Nadkarni *et al*., 2017) in other programmes.

### Procedures

#### Identification and recruitment

In the community setting, participants were identified through self-referrals (patients contacting the project staff) after coming across project leaflets distributed in the community. In the hospital outpatient department and in the PHCs/CHCs, participants were identified through: (a) universal screening of patients attending the services, (b) referrals from medical officers or other hospital staff, (c) self-referral and (d) screening conducted during awareness events such as health camps conducted to improve awareness about mental health. None of the persons visiting these facilities were seeking mental healthcare (as these services were not available). People visiting these health facilities for issues related to general health conditions along with their family members/caregivers were approached by the research team. Recruitment of participants was not limited to the patients visiting the facilities but everyone who was in the waiting areas of the OPDs (including both patients and family members) was approached for screening. Those who consented were screened using GHQ-12 and AUDIT. Among this population, those who screened positive and consented to participate in the study were recruited. Sometimes a participant who had screened negative on GHQ-12 was still referred to the psychiatrist for specialist assessment if in the judgement of the counsellor there was an issue worth exploring further. As GHQ-12 is a screening tool, it can miss out certain aspects of mental health conditions which become apparent only through narrative clinical history. A further decision on treatment continuation or discharge was taken by the psychiatrist based on their clinical assessment. If the psychiatric assessment indicated presence of a mental health condition, the participant was recruited into the programme.

#### Intervention

Following the recruitment of eligible and consenting participants, each patient was provided with the first assisted tele-consultation session of 20–30 min duration with the psychiatrist delivered through the customised online platform. The lay counsellor would conduct a brief clinical assessment of the patient (covering family history, medical history and suicide risk assessment) before the session and would then brief the psychiatrist. The sessions were facilitated by the lay counsellor for use of online software and conducted in a separate room set up in facilities where only one patient was allowed in the room at a time to ensure privacy and confidentiality. The treatment plan, including the number and duration of sessions, was decided by the psychiatrist in consultation with the patient and the lay counsellor. A medicine prescription (if required) would be shared by the psychiatrist through the online platform and a printed copy was given to the patient by the counsellor. Medicines were procured by the patients through facility pharmacies or private pharmacies (in case medicines were not available in the facilities). Family members played an important role in the treatment as they were involved at every step including the sessions (with consent from participants), getting medications, and liaising with the lay counsellors. The lay counsellors were trained to monitor the patient's pulse, blood pressure and weight which were recorded during every session. The psychiatrist recommended physical examinations, blood tests, brain scans, etc., whenever necessary, which were arranged through the lay counsellor who liased with the medical officer in the facilities. In addition, if recommended by the psychiatrist, the lay counsellor provided face-to-face counselling services to the patients, which included client-centred supportive counselling, and a contextually appropriate evidence-based psychological treatment package for either depression (HAP) (Patel *et al*., 2017) or harmful drinking (CAP) (Nadkarni *et al*., 2017). The lay counsellors also provided visits to patients' homes to conduct sessions in situations where patients were unable to attend the facilities due to difficulties such as childcare or caring for sick persons at home.

The decision to discharge a patient from care was based on the clinical judgement of the treating psychiatrist. Patients were discharged if they achieved remission or dropped out of treatment. A dropout was defined as those patients who: (a) consented to be enrolled in the study but did not follow-up for treatment, or (b) had an unplanned discharge (dropped out after entering treatment). Patients in both cases were followed up with multiple calls (four calls, each at an interval of one week) by the lay counsellors before assigning them as drop-outs. Patients were further referred to government facilities if the psychiatrist decided that they required intensive treatment, e.g. in-patient care.

#### Tele-psychiatry platform

Using data from the formative phase, we customised an existing Electronic Medical Record (EMR) and telemedicine web application, called ‘3 AM Therapy’, to meet contextual requirements. Some of its features include appointment scheduling, video conferencing, history taking format, generating and sharing of notes and files, medicine prescription printing, and session timer. The psychiatrist's dashboard displayed a summary of upcoming sessions and completed sessions (with the respective notes made during previous sessions). The web application carried minimal images to facilitate its usage even with low Internet bandwidth, was compatible with both computer and mobile devices, and its functionality allowed the patients and the doctor to rejoin an ongoing session in case it was disrupted due to power outage, disruption of connectivity or any other reason.

### Data

At baseline, we collected the following data:

- Sociodemographic information: Age, education status, employment status, gender, marital status and monthly household income.
- AUDIT: 10-item standardised screening tool developed by the World Health Organisation (WHO) to identify alcohol use disorders. A score of 16–19 indicates harmful drinking and more than 19 indicates dependent drinking (Babor *et al*., 1992).
- GHQ-12: 12-item standardised questionnaire to identify common mental disorders. A score of 4 and above indicates a possible common mental disorder (Goldberg and Blackwell, 1970; Goldberg and Williams, 1988).
- World Health Organisation Disability Adjustment Schedule 2.0 (WHODAS 2.0): A WHO tool to provide a standardised assessment of the overall functioning of the participants in the last 30 days (Üstün, 2010).

All these tools have been validated for use in India (Endsley *et al*., 2017*a*, 2017*b*; Qin *et al*., 2018) and used extensively in the study settings (Patel *et al*., 2011, 2008; Pillai *et al*., 2013). At 3 months after recruitment, we readministered GHQ-12 and WHODAS 2.0 to all patients and AUDIT (only to patients who screened positive on AUDIT at baseline) for outcome assessment.

Through the course of intervention delivery, the following data were collected:

- Patient satisfaction: After each session conducted on the online platform, patients rated the session on a bespoke satisfaction questionnaire containing parameters of audio and video quality, comfort with technology, focus of the session, overall satisfaction and willingness to use the platform again, all on five-point Likert scales (online Supplementary Material 1). The scale was developed by reviewing items from assessment tools used in 93 global tele-psychiatry-based studies (Hubley *et al*., 2016).
- Implementation process data: Measures such as the number of both tele-consultation and counselling sessions delivered, duration of tele-consultation sessions, number of patients who received different components of treatment such as tele-consultation with the psychiatrist, counselling and medicine prescription.

Serious adverse events (SAEs) for this study included hospitalisation (planned/unplanned), suicide attempt and death due to suicide or other causes. SAEs were detected either during intervention sessions, a follow-up call with the patient or during outcome assessment, and were reported to the host institution's Institutional Review Board.

### Analyses

Descriptive statistics were used to analyse the data using Stata SE 14. Socio-demographic characteristics were described as proportions and means as appropriate, for the full sample and compared between: (a) those who had planned discharge *v.* those who were dropouts, and (b) those who completed follow-up assessment *v.* those who were lost to follow-up, using χ^2^ test or *t* test, as appropriate. Paired *t* tests were used to analyse the preliminary impact of the treatment by comparing average GHQ-12 and WHODAS 2.0 scores pre- and post-treatment. The association between change in GHQ-12 and WHODAS 2.0 scores (reduced score *v.* increased/no change in score) and the socio-demographics and process indicators was calculated as odds ratios (OR) using univariate logistic regression. All variables associated with a change in these scores at *p* < 0.1 on univariate logistic regression were included in a multivariate model and variables were then excluded one by one until all remaining variables were independently associated with the outcome at *p* < 0.05.

### Ethical approval

The authors assert that all procedures involving patients were approved by the Institutional Review Board of Sangath (reference number AN_2016_019). Written informed consent was obtained from all patients. Patients with hazardous drinking were given a brief intervention. Patients with dependent drinking (who did not consent) were given a leaflet for information and were referred to the medical officers at the respective facilities. Patients who were tested positive on GHQ-12 and refused consent to participate in the study were given a leaflet containing information about signs and symptoms of general mental health problems and ways to cope. Patients who required any further treatment after the end of the study treatment were referred to government healthcare facilities for further care.

## Results

**Fig. 1.**
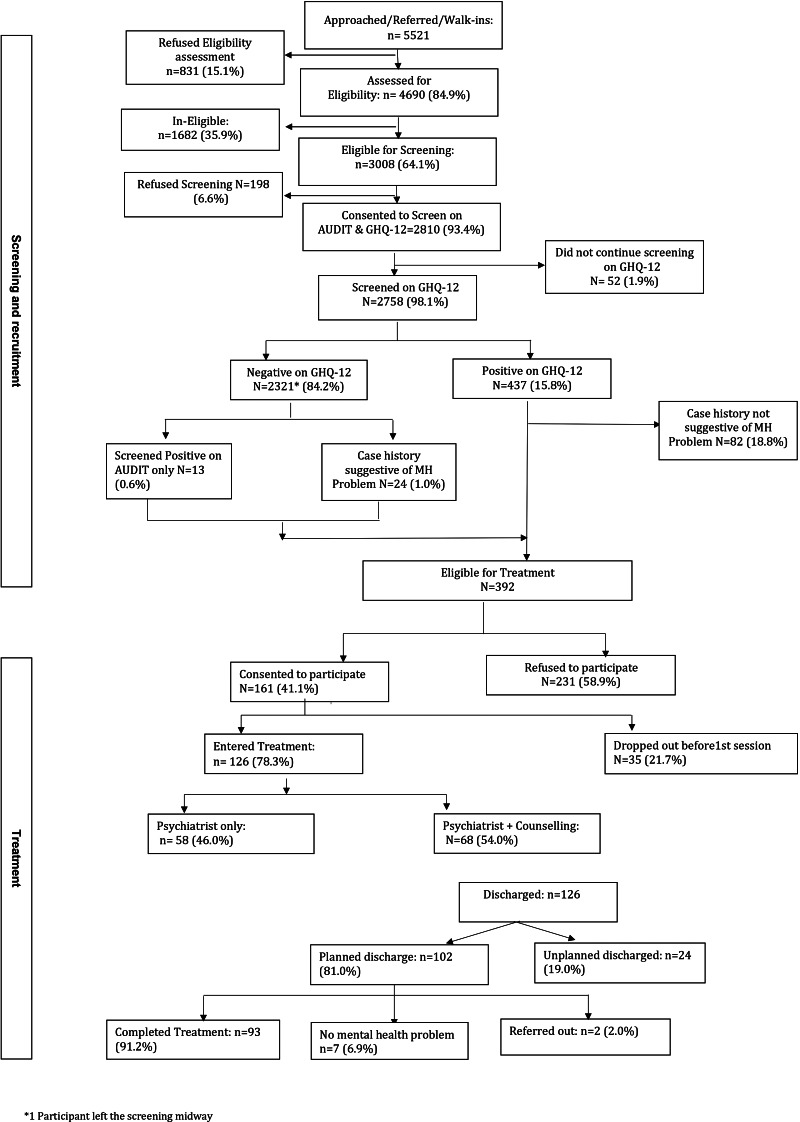
Flowchart depicting the screening and recruitment of patients recruited from all recruitment sites.

Figure 1 describes the entire process from the point of screening to discharge from treatment. A total of 161 (41%) participants (out of those eligible for treatment) consented to receive the treatment and were recruited in the study (95 from PHCs/CHCs, 60 from a public trust hospital and six from community panchayat). Majority of the 231 participants who refused consent cited reasons such as ‘no interest’ (39%), ‘no time’ (35%), ‘did not get permission from family members’ (8%) and a few other reasons (18%) including ‘do not use phones’ (3%), ‘did not follow for consent’ (3%), ‘taking treatment from elsewhere’ (2%), etc.

### Socio-demographic characteristics

The mean age of the participants was 49.6 years (s.d. 14.5) (Table 1). Majority of the participants were females (63%), married (72%), homemakers (48%) and had completed some form of education (80%). Participants who entered treatment were significantly older (51.8 years (s.d. 14.1)) compared to those who dropped out of the treatment before the first session [41.7 years (s.d. 13.4), *p* < 0.0005].

**Table 1.** Socio-demographic characteristics of all participants (*n* = 161) and participants who entered treatment (*n* = 126) *v.* those who dropped out before the first session (*n* = 35)

Characteristics	Overall (*n* = 161) *N* (%)	Entered treatment (*n* = 126) *N* (%)	Dropped out before first session (*n* = 35) *N* (%)	*p* value
Mean age in years (s.d.)	49.6 (14.5)	51.8 (14.1)	41.7 (13.4)	<0.0005
Sex				
Male	60 (37.3)	44 (34.9)	16(45.7)	0.24
Female	101 (62.7)	82 (65.1)	19 (54.3)	
Age group				
18–44 years	61 (37.9)	40 (31.7)	21 (60.0)	
45–64 years	74 (46.0)	62 (49.2)	12 (34.3)	0.006
⩾65 years	26 (16.1)	24 (19.1)	2 (5.7)	
Marital status				
Single	18 (11.2)	10 (7.9)	8 (22.9)	
Married	116 (72.0)	93 (73.8)	23 (65.7)	0.04
Separated/divorced/widow(er)	27 (16.8)	23 (18.3)	4 (11.4)	
Education status				
No formal schooling	31 (19.5)	29 (23.0)	2 (6.0)	
Completed primary	34 (21.4)	26 (20.6)	8 (24.2)	
Completed secondary school	61 (38.4)	48 (38.1)	13 (39.4)	0.20
Completed higher secondary school	15 (9.4)	10 (7.9)	5 (15.2)	
Graduate/post-graduate	18 (11.3)	13 (10.3)	5 (15.2)	
Mv	2	0	2	
Employment status				
Employed	61 (38.1)	44 (34.9)	17 (50.0)	
Unemployed/student	6 (3.7)	2 (1.6)	4 (11.8)	0.007
Retired	17 (10.6)	15 (11.9)	2 (5.9)	
Homemaker	76 (47.5)	65 (51.6)	11 (32.3)	
Mv	1	0	1	
Monthly household income				
Below Rs 10 001	39 (39.0)	30 (39.5)	9 (37.5)	
Rs 10 001 and above	61 (61.0)	46 (60.5)	15 (62.5)	0.86
Mv	61	50	11	

Mv, missing values.

### Prevalence and types of mental health problems

Of the 161 participants who consented to participate in the study, 126 entered treatment and received at least one tele-consultation session from the psychiatrist. The majority of the 126 participants who entered treatment were diagnosed by the psychiatrists as suffering from common mental disorders such as depression (35%), adjustment disorder (23%) and anxiety disorder (9%). Only 5% of participants were screened positive for harmful or dependent drinking on the AUDIT. In total, 23% of participants had other diagnoses, including dementia (2%), paranoid schizophrenia (2%), somatisation disorder (2%), mixed bipolar episodes (2%), etc. In total, 5% of participants who screened positive on the GHQ-12 were found to have no current mental disorder when assessed by the psychiatrist during the first session (online Supplementary Material 2).

### Types of treatment received

Of the 126 participants who entered treatment, 58 (46%) received only tele-consultation from the psychiatrist. Among these patients, 45 (78%) patients were also prescribed medications. A total of 68 (54%) patients received both tele-consultation from a psychiatrist and counselling from a lay counsellor (Fig. 1). Among these, 28 (41%) patients also received a medicine prescription from the psychiatrist. Out of the 68 patients who received counselling, 63% received counselling for HAP, 6% received counselling for CAP, 18% received supportive counselling sessions, 12% received both HAP and supportive counselling sessions and 1% received sessions on tobacco cessation.

### Utilisation of assisted tele-psychiatry services

A total of 626 sessions were conducted with patients comprising both assisted tele-consultation from psychiatrists (64%) and face-to-face counselling from lay counsellors (36%). Of the 402 assisted tele-consultation sessions, 71% were conducted on the 3AM therapy online platform, whereas the remaining 29% were conducted on other platforms such as Skype, WhatsApp and phone voice calls (in case of no Internet connectivity). The participants received an average of five sessions (s.d. 3.7) of tele-consultation and/or counselling. The duration of assisted tele-consultation sessions with the psychiatrist ranged from 5 to 37 min with an average duration of 8.3 min (s.d. 3.02).

### Differences between patients with planned *v.* unplanned discharge, and between those completing follow-up assessments, *v.* lost to follow-up

Overall, 102 (81% of those who entered treatment) participants had a planned discharge from the treatment. The baseline socio-demographic characteristics of those who had a planned discharge did not differ significantly compared to those who had an unplanned discharge except for their education status (*p* = 0.007) as those who dropped out of treatment had higher levels of education. In total, 113 (70.2% of the 161 consenting to treatment) participants completed their outcome assessment. The majority of those lost to follow-up (overall 30%) could not be contacted (50%), were not interested or did not have time (27%), could not keep appointments after multiple follow-ups (8%), withdrew consent (4%), moved to other places, were hospitalised or died (2%), or could not understand the questions and hence were deemed lost to follow-up (2%). There was no statistically significant difference in baseline characteristics between those who completed the outcome assessments and those who were lost to follow-up (online Supplementary Material 3).

### Clinical outcomes

There was a statistically significant reduction in both the mean GHQ-12 and mean WHODAS 2.0 scores between baseline and follow-up. Only seven patients who entered treatment were diagnosed with alcohol use disorders, and only six provided follow-up data, hence although AUDIT scores did reduce markedly, the numbers are too small to draw conclusions (Table 2).

**Table 2.** Clinical outcomes of the patients who received treatment in IMPACT

Clinical measures	Point of measurement	Baseline and outcome scores		
		Mean (s.d.)	95% Confidence intervals	*p* value
GHQ-12 (*n* = 110)^a^	Baseline	6.31 (2.44)	5.85–6.77	<0.001
	Outcome	3.31 (2.81)	2.78–3.84	
WHODAS 2.0 (*n* = 111)^a^	Baseline	23.77 (9.25)	22.03–25.52	<0.001
	Outcome	18.12 (6.45)	16.90–19.33	
AUDIT (*n* = 6)^a^	Baseline	21.50 (2.35)	20.66–26.59	0.001
	Outcome	9.83 (4.17)	5.46–14.21	

a Number of participants for whom data available for both baseline and 3-month post baseline.

On multivariate logistic regression, a reduction in GHQ-12 score was associated only with being employed or a homemaker. Reduction in WHODAS 2.0 scores was not associated significantly with socio-demographic and service utilisation variables (Table 3).

**Table 3.** Socio-demographics and other correlates of changes in GHQ-12 scores and changes in WHODAS 2.0 scores from baseline to outcome

	Change in GHQ-12 score				Change in WHODAS 2.0 score	
Variables	OR (95% CI) Univariate (*n* = 110)	*p* value	OR (95% CI) Multivariate (*n* = 109)	*p* value	OR (95% CI) Univariate (*n* = 110)	*p* value
Age						
⩽50 years	1				1	
>50 years	0.45 (0.17–1.19)	0.11			0.53 (0.22–1.24)	0.14
Gender						
Female	1				1	
Male	0.70 (0.27–1.85)	0.48			0.65 (0.27–1.57)	0.35
Employment status						
Unemployed	1		1		1	
Employed	7.19 (1.79–28.89)	0.005	8.74 (1.92–39.75)	0.005	1.94 (0.55–6.77)	0.30
Homemaker	5.70 (1.55–21.03)	0.009	6.42 (1.61–25.57)	0.008	2.93 (0.84–10.22)	0.09
Mv	1		9			
Education status						
No formal schooling	1				1	
Primary education and above	0.79 (0.24–2.63)	0.71			1.18 (0.45–3.08)	0.73
Mv = 1						
Relationship status						
Single, separated, divorced, widow(er)	1				1	
Married or in a relationship	1.36 (0.47–3.96)	0.57			1.18 (0.45–3.08)	0.73
Monthly income						
⩽Rs.10 000	1				1	
>Rs.10 000	0.67 (0.19–2.45)	0.55			0.79 (0.27–2.36)	0.68
Mv	45				44	
Type of treatment						
Only psychiatric consultations	1				1	
Only counselling	0.58 (0.06–5.60)	0.64			1.33 (0.22–8.10)	0.75
Only medication	0.33 (0.04–3.06)	0.33			0.60 (0.11–3.36)	0.78
Medication and counselling	0.5 (0.05–4.98)	0.55			0.78 (0.13–4.67)	
Mv	9				8	
Number of TP sessions						
⩽3 sessions	1		1		1	
>3 sessions	0.39 (0.15–1.05)	0.06	0.41 (0.14–1.17)	0.10	0.55 (0.22–1.37)	0.20
Mv	9				8	
Satisfaction with TP						
Dissatisfied with TP	1				1	
Satisfied with TP	0.64 (0.23–1.73)	0.37			0.68 (0.27–1.71)	0.41
Mv					13	
Change in WHODAS 2.0 score						
No change/increased score	1	0.22				
Reduced score	1.89 (0.69–5.14)					
Mv	2					
Change in GHQ-12 score						
No change/increase in GHQ-12 score					1	
Reduction in GHQ-12 score					1.89 (0.69–5.14)	0.22
Mv					3	

Mv, missing values.

### Satisfaction with the services

The average satisfaction scores for the online assisted tele-consultation sessions showed that the patients were ‘satisfied’ (score = 4) with the audio and video quality of the platform. The participants said that they could focus ‘well’ (score = 4) on the session without distraction from the technology platform and rated the functioning of the video system as ‘good’ (score = 4). On average, participants were ‘comfortable’ (score = 4) with the videoconferencing technology and were ‘willing’ (score = 4) to use telemedicine again in the future. There was no statistically significant difference in the patient satisfaction scores of those who completed the treatment *v.* those who were lost to follow-up.

## Discussion

In this study, we examined the preliminary impact and acceptability of delivering assisted tele-psychiatry in primary care settings. Since we approached individuals who had presented to health care settings for issues related to physical health conditions for either themselves or their family members, i.e. they were not seeking mental health care when invited to participate in the research project, they were met with a reluctance to participate in this project. This was reflected in the observed high rate of refusal (approximately 60%) among those who screened positive and were eligible for treatment. Coupled with high levels of stigma towards mental illness and unfamiliarity towards tele-psychiatry, this refusal rate is understandable. We observed that the majority of the participants who were recruited in the programme and diagnosed by the psychiatrist suffered from common mental disorders. We found a significant reduction in both the mean GHQ-12 score and the mean WHODAS 2.0 score between baseline and 3-month outcome assessment. We also found that a change in GHQ-12 score between baseline to follow-up was associated with employment status. Our study findings suggest that patients receiving assisted tele-consultations from the psychiatrist through the online platform achieved high levels of satisfaction with the treatment and the online platform.

There were wide variations in the duration and number of tele-consultation sessions delivered to the patients. A possible reason for this being that the management plan for each patient was customised to the needs of the individual patient as would happen in a face-to-face clinical encounter in the real world. Hence, the duration and number of sessions depended upon factors such as patients' ability to report clinical history, need to take inputs from family members, requirement of delivering counselling-related inputs/advise, reassurance when distressed, clinical needs, etc. These variations resulted in the variability in the number and duration of sessions.

Tele-psychiatry can be used to reliably diagnose a wide range of psychiatric disorders such as depression, anxiety, cognitive decline and psychosis, with a high degree of interrater reliability (Hilty *et al*., 2004). It is at par with face-to-face clinical interactions for patient satisfaction (García-Lizana and Muñoz-Mayorga, 2010; Tarp *et al*., 2017), patient/provider interaction, cultural competence, communication, trust and confidentiality (Shore *et al*., 2008). Overall, it is acceptable to patients and allows the building of relationships, serving to empower patients, providers, programmes, and communities (Hilty *et al*., 2004). Additional benefits for patients include decreased travel time and travel-related costs, none or decreased time off work, decreased waiting times, early diagnosis and intervention, better treatment adherence, and less stigma (Doze *et al*., 1999; Hilty *et al*., 2004; De Las Cuevas *et al*., 2006; Goel, 2011). Health system benefits include avoidance of hospitalisation and savings to the health system with more productive use of psychiatrists' time and availability of more consultation time (Hilty *et al*., 1999; Hailey *et al*., 2002; García-Lizana and Muñoz-Mayorga, 2010) However, the majority of the existing evidence for tele-psychiatry studies has emerged from high-resource settings and minimal evidence exists in low-resource settings (Acharibasam and Wynn, 2018), making this study on tele-psychiatry's acceptability and preliminary effectiveness all the more necessary (Naskar *et al*., 2017). Although our study did not collect quantitative data to support this, we know for a fact that people who had never received any mental healthcare before were able to receive care due to IMPACT. In the absence of IMPACT, even if their mental health problems were detected, they would have had to travel long distances at considerable cost to themselves to the nearest mental healthcare provider. Hence, we can make a reasonable assumption that receiving mental health care closer to their homes would result in time and cost savings for the patients and their families.

Our treatment integrated tele-consultation from the psychiatrist (assisted by counsellors) with counselling from lay counsellors, along with medication prescription (if required), creating a holistic package of care. The majority of previous studies have compared tele-psychiatry with face-to-face treatment and reported tele-psychiatry to attain superior or equivalent outcomes (Ruskin *et al*., 2004; Moreno *et al*., 2012). Our study attempted to integrate assisted tele-psychiatry services within primary care settings in remote, under-resourced areas and in a naturalistic environment. The focus of this study was in keeping with the spirit of the NMHP of India, i.e. to make appropriate and effective mental health services available and accessible at the grassroots level (Ministry of Health & Family Welfare, 2014).

Our findings reflect a high level of satisfaction with the online platform in terms of quality of audio-visual components, comfort with the online platform and willingness to continue treatment on the platform. These findings are consistent with those reported in a review by Hubley et al. in 2016 where the majority of the studies rated tele-psychiatry services as ‘good’ to ‘excellent’ (Hubley *et al*., 2016). Similar high patient satisfaction has been reported in various literature and systematic reviews conducted globally, where treatment delivered through videoconferencing has been found to be equivalent or superior to face-to-face treatment (Chakrabarti, 2015; Hubley *et al*., 2016). These findings are indicative of the level of acceptability of the platform among patients, making the online platform an appealing option to access services.

### Implications for research, policy and practice

Our findings have demonstrated that treatment delivered to people experiencing mental disorders through an assisted tele-psychiatry model, in primary care settings, attains a high patient satisfaction rate. This will add to the evidence-base of the possible integration of assisted tele-psychiatry to deliver mental healthcare in primary care in low-resource settings.

With the advent of the global COVID-19 pandemic and the resulting need for physical distancing, tele-psychiatry has emerged as a viable alternative to in-person care (Perera *et al*., 2020). The benefits of tele-psychiatry, including access to care at the patient's own home, reduced travel time and expenses, and easy access to specialist care, have been especially realised in the wake of the pandemic (Agarwal *et al*., 2020). Realising its potential in healthcare delivery, the Medical Council of India (MCI) adopted the ‘Telemedicine Practice Guidelines’ in March 2020 to assist medical professionals to practice telemedicine with a sound course of action and provide safe and effective care (Medical Council India, 2020). This is a positive step towards the adoption of telemedicine and the use of its application in psychiatry (tele-psychiatry), for providing regular care to patients and to cope with the current pandemic.

### Strengths and limitations

One of the major strengths of our study is its scope and aims. There is limited research regarding the integration of assisted tele-psychiatry in primary care, especially in a low-resource setting such as India. Although tele-psychiatry does not create more mental health resources, it does help in efficient redistribution of resources. Thus, integration of tele-psychiatry in the primary health care systems can help solve the ‘accessibility’ problem. However, we do acknowledge that this can only work sustainably if we continue to build human resources and ensure equitable access to digital technology. Additionally, our study explored the delivery of a holistic care package including counselling from lay counsellors and the provision of a medication prescription. We effectively used the strategy of ‘task sharing’ to make ‘available’ quality and evidence-based psychological treatment via trained lay counsellors. These lay counsellors belonged to the same social fabric as the service users and understood the local context in which the mental health problems existed. It brought down the barriers to seeking help and contributed to high levels of client satisfaction. Other major strengths of our study include the use of standardised assessment tools and questionnaires and recruitment from multiple settings, giving a wider perspective and allowing for greater generalisation. The limitations of our study include the lack of a comparison group, a short follow-up period of 3 months and self-report assessment. There was potential response/acquiescence bias while recording the patient satisfaction scores. However, we implemented a few mitigation strategies including inclusion of a five-point Likert scale instead of simple binary responses and self-administration of questionnaire instead of researcher administration to reduce the bias. Another limitation of the study is that we did not collect data on the provider's satisfaction with this new modality for delivering treatment.

## Conclusion

In conclusion, treatment in low-resource settings of mental health problems through a tele-psychiatry platform appears to be highly acceptable and is associated with improved clinical outcomes. These preliminary results need to be corroborated via a full randomised controlled trial, and considering its potential for scalability, a model of assisted tele-psychiatry integrated into primary care can be an important strategy to increase access to mental healthcare in low-resource settings.

## Supplementary material

For supplementary material accompanying this paper visit http://dx.doi.org/10.1017/gmh.2021.47. click here to view supplementary material
