# Supplementary material for: Integrating assisted tele-psychiatry into primary healthcare in Goa, India: a feasibility study
Source: Glob Ment Health (Camb). 2022 Feb 3;9:26–36. doi: 10.1017/gmh.2021.47 (PMC9806979; doi:10.1017/gmh.2021.47)
Supplement: Supplementary file 1 [file S2054425121000479sup.zip › S2054425121000479sup001.pdf]

Supplementary material 1

Tele-psychiatry Session Completion Questionnaire (To be completed after each session)

Below you will find some questions regarding your experience in today's tele-psychiatry session.

Please keep in mind, these questions are not about the psychiatrist you spoke with, but about the videoconferencing technology you used.

As such, please answer these questions thinking about the videoconferencing technology only:

a) How would you rate your satisfaction with the sound quality of today's session?

Very Dissatisfied	Dissatisfied	Neutral	Satisfied	Very Satisfied
1	2	3	4	5

b) How would you rate your satisfaction with the picture quality of today's session?

Very Dissatisfied	Dissatisfied	Neutral	Satisfied	Very Satisfied
1	2	3	4	5

c) Overall, how would you rate the functioning of the video system?

Very Poor	Poor	Neutral	Good	Very Good
1	2	3	4	5

d) How well were you able to focus on what was being said today without being distracted by the videoconferencing technology?

Not Well At All	Not Well	Neutral	Well	Very Well
1	2	3	4	5

e) How comfortable did you feel with the video conferencing?

Very Uncomfortable	Uncomfortable	Neutral	Comfortable	Very Comfortable
1	2	3	4	5

f) After this interview, how willing are you to use telemedicine again?

Not Willing At All	Not Willing	Neutral	Willing	Very Willing
1	2	3	4	5

g) Did today's session meet your expectations?

Yes　　　　No

Please briefly explain:

____

____

____

____

This is the end of the Session Completion Questionnaire. Thank you for your feedback.
