# Supplementary material for: Integrating assisted tele-psychiatry into primary healthcare in Goa, India: a feasibility study
Source: Glob Ment Health (Camb). 2022 Feb 3;9:26–36. doi: 10.1017/gmh.2021.47 (PMC9806979; doi:10.1017/gmh.2021.47)

Supplementary material 2

Figure: Distribution of diagnosis of the patients who received treatment from IMPACT (n= 126)

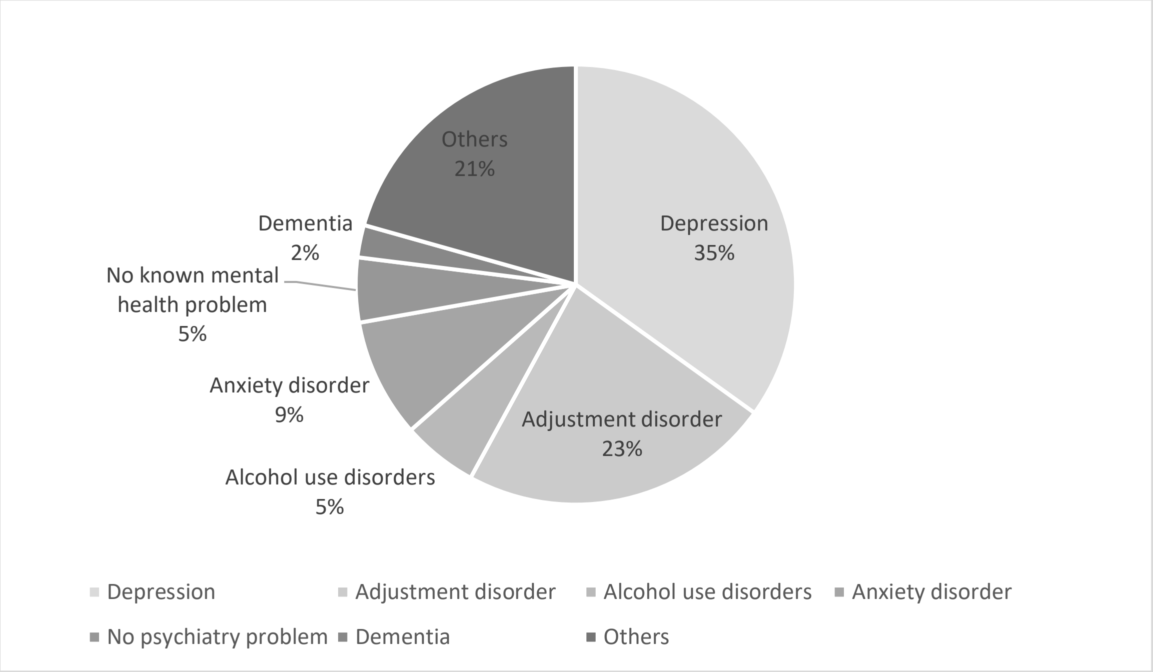

Supplement: Supplementary file 1 [file S2054425121000479sup.zip › S2054425121000479sup002.pdf]
