# Supplementary material for: Integrating assisted tele-psychiatry into primary healthcare in Goa, India: a feasibility study
Source: Glob Ment Health (Camb). 2022 Feb 3;9:26–36. doi: 10.1017/gmh.2021.47 (PMC9806979; doi:10.1017/gmh.2021.47)
Supplement: Supplementary file 1 [file S2054425121000479sup.zip › S2054425121000479sup003.pdf]

Supplementary material 3

Table: Socio-demographic characteristics of patients with respect to their discharge status and completion of outcome assessment (N=161)

**Characteristics**	Patients who completed treatment/ planned discharge (n=102) N (%)	Patients who did not complete treatment/ unplanned discharge (n=24) N (%)	p-value	Patients who completed outcome assessments (n= 113) N(%)	Patients who did not complete outcome assessments (n=48) N(%)	p-value
**Mean age in years (SD)**	51.53 (14.24)	53.13 (13.71)	0.62	50.53 (14.13)	47.5 (15.35)	0.22
**Sex**						
Male	34 (33.3)	10 (41.7)	0.44	38 (33.6)	22 (45.8)	0.14
Female	68 (66.7)	14 (58.3)		75 (66.4)	26 (54.2)	
**Age group**						
18-44 years	31 (30.4)	9 (37.5)	0.43	38 (33.6)	23 (47.9)	
45-64 years	53 (52.0)	9 (37.5)		55 (48.7)	19 (39.6)	0.23
≥65 years	18 (17.6)	6 (25.0)		20 (17.7)	6 (12.5)	
**Marital status**						
Single/separated/divorced/widow(er)	27 (26.5)	6 (25.0)		28 (24.8)	17 (35.4)	0.17
Married	75 (73.5)	18 (75.0)	0.88	85 (75.2)	31 (64.6)	

**Education status**						
No formal schooling	22 (21.6)	7 (29.2)		23 (20.5)	8 (17.0)	
Completed Primary	24 (23.5)	2 (8.3)	0.007	22 (19.6)	12 (25.5)	
Completed secondary school	41 (40.2)	7 (29.2)		43 (38.4)	18 (38.3)	0.56
Completed higher secondary school	9 (8.8)	1 (4.1)		9 (8.1)	6 (12.8)	
Graduate/Post-graduate	6 (5.9)	7 (29.2)		15 (13.4)	3 (6.4)	
Mv	0	0		1	1	
**Employment status**						
Employed	35 (34.3)	5 (20.8)	0.41	43 (38.4)	18 (37.5)	
Unemployed/student/retired	12 (11.8)	9 (37.5)		14 (12.5)	9 (18.8)	0.57
Homemaker	55 (53.9)	10 (41.7)		55 (49.1)	21 (43.7)	
Mv	0	0		1	0	
**Monthly household income**						
Below Rs10001	22 (37.9)	8 (44.4)	0.62	26 (38.2)	13 (40.6)	0.82
Rs 10001 and above	36 (62.1)	10 (55.6)		42 (61.8)	19 (59.4)	
Mv	44	17		45	16	

MV= Missing values
